# Absorbed dose calculations in a brachytherapy pelvic phantom using the Monte Carlo method

**DOI:** 10.1120/jacmp.v3i4.2552

**Published:** 2002-09-01

**Authors:** Miguel L. Rodríguez, Carlos E. deAlmeida

**Affiliations:** ^1^ Laboratório de Ciências Radiológicas Universidade do Estado do Rio de Janeiro Rua São Francisco Xavier, 524 Maracanã Rio de Janeiro RJ Brazil

**Keywords:** Brachytherapy, Monte Carlo applications, simulation, and phantoms

## Abstract

Monte Carlo calculations of the absorbed dose at various points of a brachytherapy anthropomorphic phantom are presented. The phantom walls and internal structures are made of polymethylmethacrylate and its external shape was taken from a female Alderson phantom. A complete Fletcher‐Green type applicator with the uterine tandem was fixed at the bottom of the phantom reproducing a typical geometrical configuration as that attained in a gynecological brachytherapy treatment. The dose rate produced by an array of five ^137^Cs CDC‐J type sources placed in the applicator colpostats and the uterine tandem was evaluated by Monte Carlo simulations using the code penelope at three points: point A, the rectum, and the bladder. The influence of the applicator in the dose rate was evaluated by comparing Monte Carlo simulations of the sources alone and the sources inserted in the applicator. Differences up to 56% in the dose may be observed for the two cases in the planes including the rectum and bladder. The results show a reduction of the dose of 15.6%, 14.0%, and 5.6% in the rectum, bladder, and point A respectively, when the applicator wall and shieldings are considered.

PACS number(s): 87.53Jw, 87.53.Wz, 87.53.Vb, 87.66.Xa

## INTRODUCTION

The goal of gynecological brachytherapy is the placement of radioactive sources in the uterus and the vagina in order to maximize the dose delivered to the tumor and minimize the dose delivered to the surrounding normal tissues. There are several factors that make it difficult to reach this purpose: the rapid dose fall‐off near the sources, difficulties in localizing the tumor and normal organs, and inconsistencies in the methods employed for dose calculation. Despite these factors, important improvements have been observed in the last few decades in the standardization of the source strength specification^1^ ^,^ ^2^ and some consensus in the aspects related to the definition of relevant clinical points.^3^

Nowadays, low dose rate Cs137 sources and high dose rate Ir192 sources of various designs, and a number of different applicators, are currently in use in gynecological brachytherapy.^4^ ^–^ ^6^ In contrast, the applicator heterogeneities are normally ignored by many commercially available treatment planning systems. The establishment of a quality assurance protocol to guarantee the desired treatment accuracy is at present mandatory, and as a part of it, the verification of the accuracy in the dose calculation of the treatment planning programs is an important task.^7^ The methods implemented to test these programs often make use of standard data sets and phantoms and compare the programs results with the values expected for those standards.

Several methods have been employed to assess the absorbed dose near brachytherapy applicators and sources.^8^ ^–^ ^10^ In contrast to measurements, the Monte Carlo dose estimates are not affected by errors in detector positioning, detector energy, angular dependence, and steep dose gradients near the sources.^11^

This article describes the use of the penelope Monte Carlo method to calculate the absorbed dose at relevant points inside of a pelvic phantom. The phantom was designed to be part of quality assurance and training programs in brachytherapy of gynecological tumors.

## METHODS

### A. Phantom description

**Figure 1:**
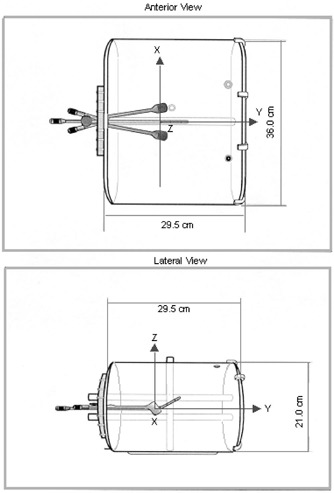
The anthropomorphic pelvic phantom and the coordinate system used for the Monte Carlo simulation.

An anthropomorphic polymethylmethacrylate (PMMA) phantom, shown in Fig. 1, has been designed with the external shape taken from a female Alderson phantom. The lengths of its major axes are: 21 cm in the antero‐posterior direction, 36 cm in the lateral direction, and 29.5 cm in the axial axis. The wall thickness is 0.5 cm.

Various inserts are placed into the phantom to allow the positioning of a cylindrical ionization chamber at the coordinates of points of clinical interest corresponding to the rectum, bladder, and point A. The material of these inserts is also PMMA, the internal diameter matches the 0.69 cm^3^ Farmer type ionization chamber, and its wall thickness is 1.2 mm.

A Fletcher‐Green type applicator^12^ ^,^ ^13^ and a uterine tandem were glued to a PMMA disk that was fixed at the bottom face of the phantom, allowing the applicator and tandem to be positioned in typical gynecological treatment geometry.

### B. Monte Carlo calculations

The absorbed dose at the clinical points was calculated by using the Monte Carlo code penelope to simulate the radiation transport in the phantom. The characteristics of the penelope code have been discussed in detail elsewhere,^14^ hence, just a brief description is provided here. The code is implemented in fortran 77 and its structure is based on a set of subroutines that are invoked from a main program written by the user. It is applicable to energies ranging from 1 keV to 1 GeV for photons and from 0.1 keV to 1 GeV for electrons. The code simulates incoherent scattering, coherent scattering, and Bremsstrahlung x‐ray production. The electron binding effects and Doppler broadening are taken into account for incoherent scattering in addition to the characteristic *k*‐shell x‐rays and Auger electron emission following photoelectric absorption. Electron and positron histories are generated on the basis of a mixed algorithm that combines detailed simulation of hard events with condensed simulation of soft events. The package for geometry definition is based on the combination of surfaces (represented by quadrics functions) to form more complex structures, such as bodies and body sets (modules).

### C. Modeling the source and phantom geometries

The radioactive Cs137 sources used in the simulation are the CDC‐J type made by Amersham International. These sources consist of cesium bound with a low attenuation ion exchange medium of zirconium phosphate with $1.63g/{\text{cm}}^{3}$ of density.^15^ The source encapsulation is 1 mm thick of a material of mass composed of 80% platinum and 20% iridium. The source dimensions are 20 mm long and 2.65 mm in diameter. In penelope the sources were modeled as a pair of concentric cylinders with dimensions and materials as described above. The phantom shape and its internal structure were modeled as filled with liquid water (density of $1g/{\text{cm}}^{3}$). The PMMA phantom wall was not modeled since its presence did not perturb the calculated dose distribution near the reference points.

**Figure 2:**
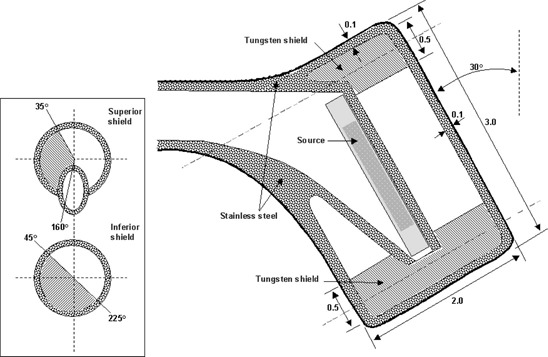
Internal structure of the colpostat. The box at left shows transversal planes taken along the shieldings (not specified dimensions are in cm).

The internal structure of the applicator was modeled following the dimensions and materials shown in Fig. 2. The applicator is a Fletcher‐Green type, its external wall is 1 mm thickness of stainless steel. The colpostats were cylindrically shaped with a 20 mm diameter, 30 mm long, angulated 30° in relation to the handle. Its internal tungsten shields are not completely cylindrical along the transversal plane of the colpostat; instead, in that plane, the shields are observed as circles extending from 35° to 160° in the superior shield and from 45° to 225° in the inferior shield (Fig. 2). The uterine tandem was simulated as a linear succession of right cylinders. Where the cylinders abutted, a slight bend was introduced to mimic the 15° bend in the tandem geometry.

**Figure 3:**
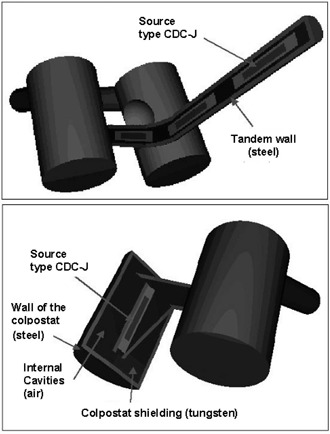
3D rendered views of the geometry of the applicator and tandem created for simulation. Cuts have been made at planes containing the sources in order to illustrate as they are inserted in the colpostats and tandem.

Figure 3 shows 3D rendered views of the simulated geometry of the applicator and tandem. In these views, cuts have been made at the planes containing the sources. The coordinate system used for the simulation was taken as shown in Fig. 1, with the origin lying at the mid‐height of the colpostats and the mid distance between them. All the internal distances from the applicator to the relevant clinical points in this coordinate system are known and defined in Table I.

### D. Dose calculation

For the dose calculation using the Monte Carlo method, the charged particle equilibrium was assumed to exist,^16^ and the absorbed dose was approximated to the collision kerma. This assumption is valid as long as the dose contribution due to the electrons generated in the colpostats and tandem structure is neglected.

The exponential track‐length estimator^11^ was used to calculate the collision kerma. For this purpose a subroutine named TRLEN was written in fortran 77, which perform ray‐tracing along the photon trajectory between collisions.

Scoring volumes with radii from 1 to 1.5 mm were used for average source‐point distances ranging from 1.5 to 3.75 cm as recommended by Williamson.^11^ The photon linear attenuation and mass energy‐absorption coefficients were taken from Hubbell and Seltzer.^17^

Five sources with a geometry as described above, were simulated, all together, placed in the colpostats and tandem in positions defined from two orthogonal x‐ray films taken with dummy sources inserted in the phantom. Two sources with a linear reference air kerma rate of $72.3\mu {\text{Gyh}}^{-1}\;m^{2}\;{\text{cm}}^{-1}$ were simulated as inserted in the colpostats and three sources with an air kerma rate of 54.2, 36.2, and $36.2\mu {\text{Gyh}}^{-1}\;m^{2}\;{\text{cm}}^{-1}$ were simulated as inserted in the uterine tandem.

In order to evaluate the applicator influence to the absorbed dose, two geometric models of the phantom were considered: one including the applicator and tandem filled with the sources, and a second one including just the sources, arranged in the same way as if they were inside the applicator and tandem.

For each model, the dose calculation was performed for the three orthogonal planes, selected in such a way that each of them contains one or more points of interest. This approach allows us to calculate the dose at these points, evaluate the isodose curves produced by the sources, and also evaluate the change produced in these curves for the applicator walls and shieldings.

In the coordinated system described above, the planes are: x=0,z=0.5cm, and y=0. Each of them comprise a region of $10\times 10{\text{cm}}^{2}$ and are symmetrically situated in relation to the center of coordinate, except for the plane x=0, which extends from −3to+7cm along the *y* axis.

For each configuration (with and without the applicator), a set of five simulations, with a total of $2\times 10^{8}$ photon histories each, was performed.

## RESULTS AND DISCUSSION

### A. Dose planes

**Figure 4:**
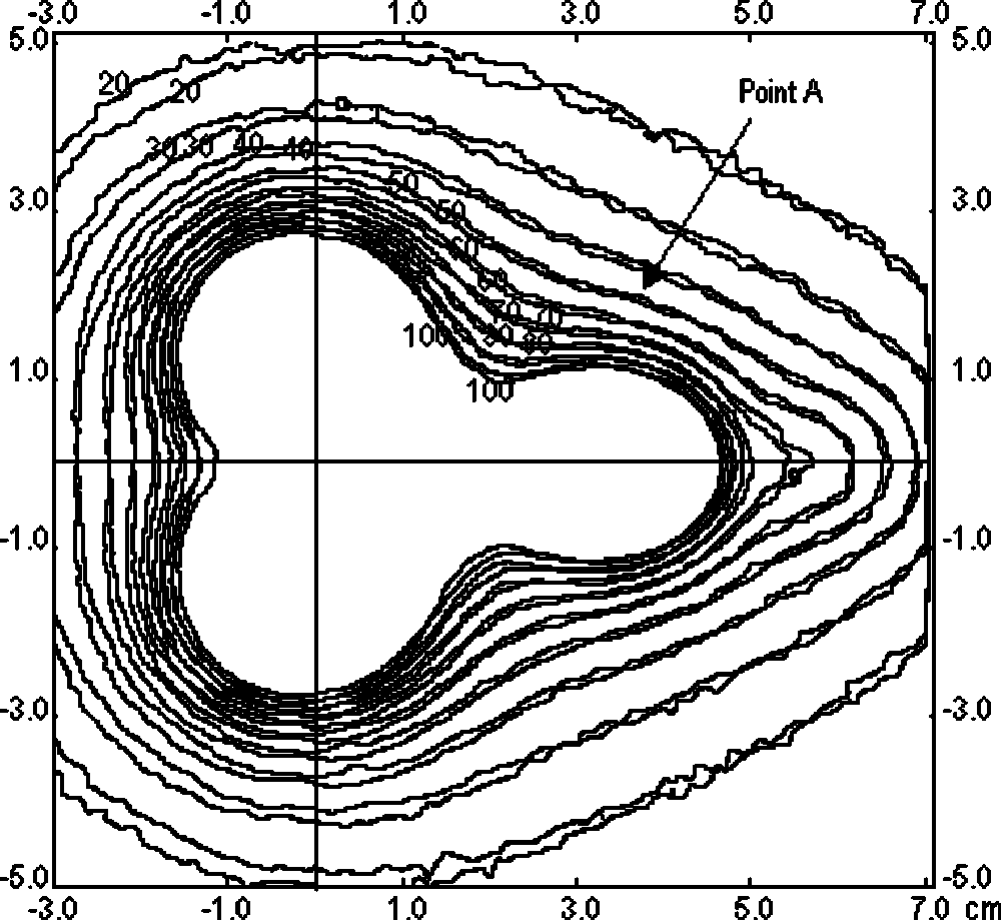
The isodose curves at plane z=0.5cm. The dose rate values are in cGy/h.

Figure 4 shows the isodose curves obtained for the plane z=0.5cm, which contains point A. The isodoses for the two models adopted are plotted together in order to evaluate the effect of the applicator wall on these curves. It may be observed that in this plane, the influence of the applicator shielding is not very significant because the region included is not primarily irradiated by photons transmitted through the shieldings. This is expected, taking into account the fact that this is a treatment region.

**Figure 5:**
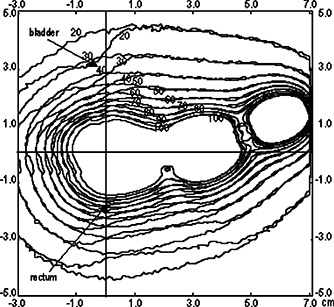
The isodose curves at plane x=0. The dose rate values are in cGy/h.

**Figure 6:**
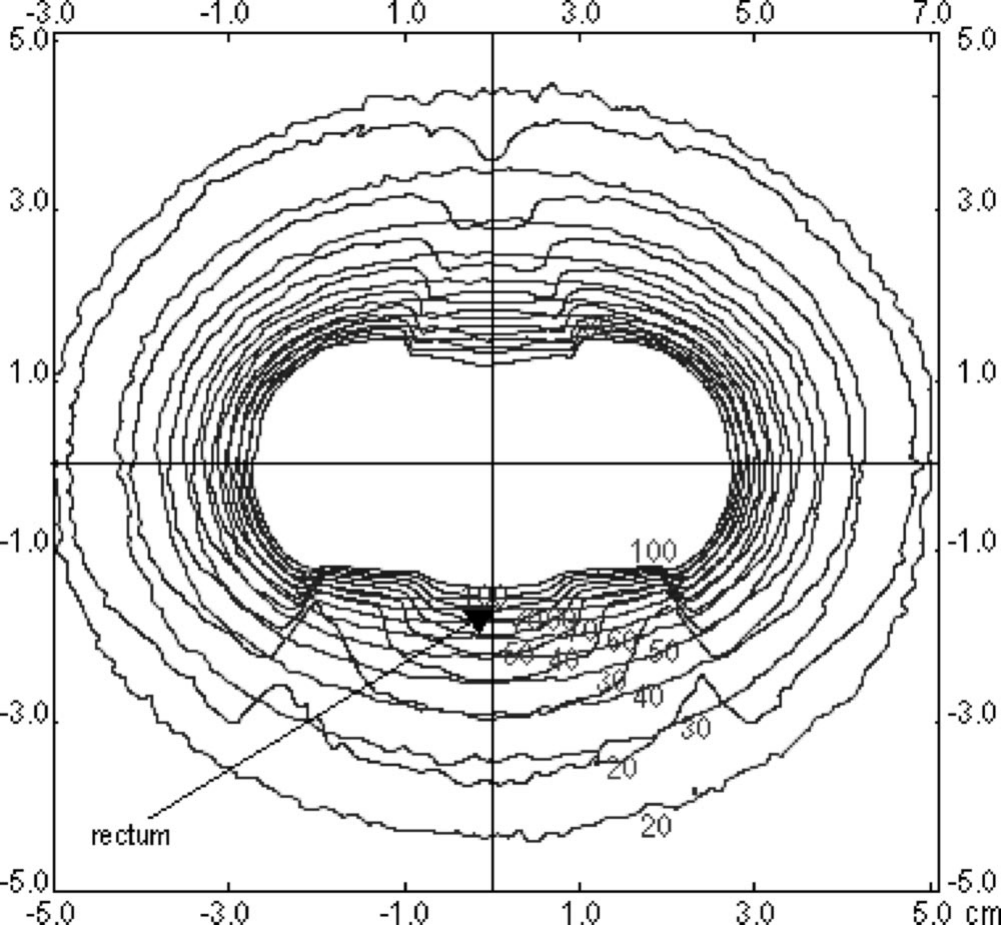
The isodose curves at plane y=0. The dose rate values are in cGy/h.

The isodoses in the plane x=0, which contains the points of rectum and bladder, are shown in Fig. 5. In this region, a considerable change in these curves is clearly observed near the applicator. The maximum change in the dose is in the zones located in the superior and inferior faces of the colpostats. This is a plane taken at the middle distance between the colpostats; therefore, the effect observed is due to the contribution of both. The maximum relative difference in the calculated dose with and without the applicator found in this plane was 42%. The same effect can be observed in the plane y=0, which contains the point of rectum (Fig. 6). As expected, the regions located above and below the colpostats present the main variation on the dose. The relative difference calculated for all points in this plane, located farther than 0.5 cm from the applicator wall, reaches a maximum of 56%.

**Table I:** Locations of the relevant point of interest relative to the system of coordinates defined for simulation.

Point of interest	*X* (cm)	*Y* (cm)	*Z* (cm)
Point A	−2.1	3.75	0.5
Rectum	0.0	0.0	−1.5
Bladder	0.0	−0.4	3.1

### B. Dose calculation at the points of interest

The absorbed dose at the prescription point (point A), the rectum, and the bladder were calculated separately and are shown in Table II for the two simulated models with the results expressed for 3*σ* with a mean standard error less than 1%.

For the location of these points in the phantom, the combined effect in the dose variation caused by the applicator walls and shielding are 15.6% for the rectum, 14.0% for the bladder, and 5.6% for point A.

The observed overestimation on the calculated dose at the points of interest is caused by ignoring the presence of the applicator by most of the commercial treatment planning systems. The algorithms used to calculate the dose at a point are based on the superposition principle, summing the individual contribution of the sources assuming a homogenous media and just taking the source encapsulation into account.

Differences between the dose reported by those programs and the actual dose delivered during the patient treatment for the particular case considered in this work, are beyond the accepted range of error for low dose brachytherapy. Reducing these differences by taking into account the dosimetric characteristics of the applicators might help the construction of more accurate dose‐response curves for a better tumor control and complications.

**Table II:** Results of dose rate evaluated at the three clinical points by the Monte Carlo method with and without the influence of the applicator.

Site	Dose rate (cGy/h)		
	Sources only (±3σ)	Applicator (±3σ)	Ratio (sources only/applicator)
Point A	46.29±0.40	43.71±0.36	1.059
Rectum	92.89±0.73	78.43±0.40	1.184
Bladder	33.76±0.31	29.04±0.24	1.163

## CONCLUSIONS

A fortran 77 program was written to use the penelope Monte Carlo code that simulates the radiation transport inside a pelvic phantom. The implemented program also uses the data created to model the phantom internal geometry and materials, including a Fletcher‐Green type applicator, uterine tandem, and the five sources inserted into them. In this program, a special ray‐tracing subroutine was written for the evaluation of the collision kerma by means of the exponential track‐length estimator.

The absorbed dose along three representative planes inside the phantom was calculated for two geometrical configurations, which consider the presence, or the absence of the applicator. Differences up to 56% in the dose for the two cases are observed in planes, including the rectum and bladder.

It was found that the variation of dose due to the effect of the applicator is 15.6% and 14.0% in the rectum and bladder respectively and just 5.6% in the point of prescription (point A). These are approximately the levels of overestimation of the dose expected in the results provided by the brachytherapy treatment planing systems that do not include the effect of the applicator in their algorithms.
